# A Dominant Plant Species and Insects Independently and Interactively Shape Plant Community Structure and Ecosystem Function Above‐ and Below‐Ground

**DOI:** 10.1002/ece3.72742

**Published:** 2025-12-19

**Authors:** Julia N. Eckberg, Nathan J. Sanders

**Affiliations:** ^1^ Department of Ecology and Evolutionary Biology University of Michigan Ann Arbor Michigan USA

## Abstract

It is well‐established that dominant plant species and insect herbivores independently shape community structure and ecosystem function in terrestrial plant communities. Critically, few studies have assessed the combined effects of these two drivers of plant community structure and ecosystem function above‐ and below‐ground or tracked their effects over time. In this study, we factorially manipulated the dominant species *Solidago canadensis* (Canada goldenrod) and insects in an old field for 3 years and quantified their effects on plant diversity, biomass, functional traits, microclimate, and decomposition. Overall, *S. canadensis* and insect herbivores independently and interactively shaped aboveground plant biomass, richness, functional traits, microclimate, and the decomposition of soil organic matter. The biomass of the rest of the plant community was higher where *S. canadensis* was removed and where insects were reduced, in part due to the positive effect of *S. canadensis* removal on light availability and soil moisture. The highest estimated decomposition rate of soil organic matter occurred where *S. canadensis* was removed and insects were reduced. Furthermore, the community weighted mean (CWM) of multiple plant functional traits related to competition for light varied with *S. canadensis* removal and insect reduction; for example, the CWM of plant height was highest where *S. canadensis* was removed and insects were reduced. After 3 years of experimental manipulation, *S. canadensis* removal and insect reduction independently and interactively reduced the temporal stability of aboveground plant biomass but not species richness. Altogether, our results highlight the need to consider the potential interactive effects of dominant plants and insect herbivores above‐ and below‐ground to improve our understanding of their role in structuring communities and ecosystems. Furthermore, we find that over 3 growing seasons insect herbivores prevent biomass compensation following *S. canadensis* removal, emphasizing the importance of insect herbivores in mediating community and ecosystem recovery after biodiversity loss.

## Graphical abstract

Though it is well‐established that dominant plant species and insects independently shape community structure and ecosystem function in terrestrial plant communities, few studies have assessed their combined effects on plant community structure and ecosystem function above‐ and below‐ground or over time. Overall, we find that a dominant plant and insects independently and interactively shaped plant biomass, richness, functional traits, microclimate, and the decomposition of soil organic matter. Furthermore, a dominant plant and insects modified the temporal stability of plant biomass, highlighting the need to consider the potential interactive effects of dominant plants and insects through time to improve our understanding of their role in structuring communities and ecosystems.

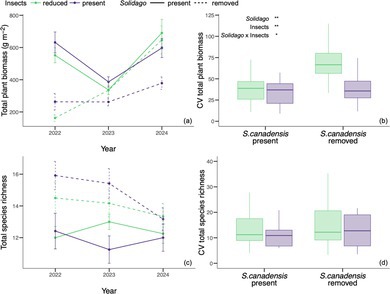

## 1 Introduction

What factors drive community structure and ecosystem function, above‐ and below‐ground, in terrestrial plant communities? One key driver is dominant plant species. Dominant plant species constitute a large proportion of biomass in a community, and as a result, shape the diversity and function of communities and ecosystems (Grime 1998; Avolio et al. 2019; Smith et al. 2020; Eckberg et al. 2025). Removal experiments reveal that dominant plants tend to reduce the richness of the plant community but increase productivity (Pinder III 1975; Mulder et al. 2004; Avolio et al. 2019; Smith et al. 2020). Dominant plants typically share a similar set of traits that lead to their competitive dominance. Dominant plants in early successional communities often have acquisitive traits (Kazakou et al. 2007; Chai et al. 2016), which allow them to maximize resource capture and outcompete other plant species for aboveground resources such as light. However, less is known about whether the presence of a dominant plant affects the traits of other plant species (but see Hejda et al. 2019).

The effects of dominant plants on communities and ecosystems also extend belowground. The Mass‐Ratio Hypothesis posits that dominant species directly alter the rate of both above‐ and below‐ground ecosystem‐level processes because of their large biomass (Grime 1998). For example, if a dominant plant species has a high transpiration rate (Kaushik et al. 2025), soil moisture could subsequently be reduced. In addition to their direct effects, dominant plants can also indirectly shape belowground processes. For instance, dominant plant species can alter the rate of soil nutrient cycling indirectly by altering plant diversity (Jiang et al. 2021). Dominant plants tend to have consistent effects on community structure and ecosystem function across studies with different focal species in a variety of ecosystems (Avolio et al. 2019). Given that the majority of communities are characterized by the presence of a few or a single dominant species (Tokeshi 1993; Avolio et al. 2019), we can apply the observed effects of a dominant plant in one system in order to predict how a different dominant species may be shaping ecological processes in a novel system. However, whether the effects of dominant plants on communities and ecosystems above‐ and below‐ground are mediated by interactions with organisms of higher trophic levels is less well understood.

Insect herbivores, like dominant plant species, are a driver of plant community structure and ecosystem function aboveground (Crawley 1989; Carson and Root 1999, 2000; Smith et al. 2020; Agrawal and Maron 2022; Eckberg et al. 2025). When insect abundance is reduced, plant species richness is lower, but plant biomass is higher (Carson and Root 1999; Smith et al. 2020). One mechanism by which insect herbivores can promote plant species richness is by reducing the aboveground biomass of highly competitive plant species (Carson and Root 2000; Kempel et al. 2015), highlighting the potential for dominant plants and insects to have unique, combined effects on plant community structure. In addition to their effects on plant richness and biomass, insect herbivores may alter plant traits. Leaf traits such as toughness and thickness are associated with herbivore resistance (Pérez‐Harguindeguy et al. 2013) and therefore likely vary in response to insect herbivory. Insect herbivores can directly affect plant traits by selectively consuming plants with thin leaves that are mechanically easier to eat (Schädler et al. 2003), which could ultimately alter community‐wide trait patterns by changing the abundance of species with particular traits or driving plastic shifts in trait expression. Vertebrate herbivores, like insect herbivores, can also shape the biomass and richness of terrestrial plant communities (Borer et al. 2014; Smith et al. 2020). Notably, vertebrate herbivores can have a negative effect on the abundance and performance on insect herbivores, particularly when they preferentially target the same plant species, suggesting that the presence of vertebrate herbivores may limit the effects of insects on plant communities (Zhu et al. 2019).

In addition to their effects aboveground, insects can also shape communities and ecosystems belowground (Hunter 2001; Classen et al. 2005, 2006). Insect herbivores can alter soil nutrient cycling directly through frass deposits or indirectly by modifying the quantity and quality of plant litter (Hunter 2001; Classen et al. 2006; Buchkowski and Schmitz 2022). The modification of plant litter quality by insects can further reshape the composition and function of the soil microbial communities that decompose plant litter and transfer nutrients through terrestrial ecosystems (Bakker et al. 2011). Through their effects on soil microbes, insects may subsequently have indirect effects on ecosystem processes such as decomposition and productivity. Insects can also modify soil microclimate (Hunter 2001; Classen et al. 2005), including soil moisture and temperature. Soil microclimate and the availability of soil nutrients can subsequently determine the diversity and productivity of plant communities (Dąbrowski et al. 2013; Lozano‐Parra et al. 2018; Smith et al. 2020), suggesting that insect herbivores have the potential to shape plant community structure and ecosystem function indirectly via their effects on belowground processes.

Importantly, the effects of dominant plants and insect herbivores on communities and ecosystems can vary over time. For example, removing a dominant plant species from the community had no effect on plant community composition until after 8 years of maintained dominant species removal (Munson and Lauenroth 2009), highlighting the importance of tracking community response to dominant species removal over multiple years. Similarly, some field experiments found that the effect of insect reduction on plant richness and biomass did not manifest until after several years of manipulation (Carson and Root 1999; Allan and Crawley 2011). Temporal lags in the effects of dominant plants and insects on plant communities could potentially shape the temporal stability of communities and ecosystems (Hillebrand et al. 2008; Grman et al. 2010; Sasaki and Lauenroth 2011; Post 2013). Previous work has established that dominant plants regulate the temporal stability of productivity (Sasaki and Lauenroth 2011; Hou et al. 2023), but the effect of insects on the temporal stability of productivity, and whether dominant plants and insects have interactive effects on temporal stability, are less well understood. As such, it is essential to track temporal trends in community and ecosystem response to dominant species removal and insect reduction.

Notably, many studies on the effects of dominant plants and insects on plant communities and ecosystem function focus on these factors in isolation (Carson and Root 1999; Souza et al. 2011; Smith et al. 2020; Hernández et al. 2022), quantify only above‐ or below‐ground responses (Carson and Root 1999; Smith et al. 2020), or evaluate their effects over short time scales (Smith et al. 2020; Hernández et al. 2022). In this study, we examine the independent and combined effects of a dominant plant species and insects on plant diversity, plant traits, resource availability, and ecosystem function above‐ and below‐ground by factorially manipulating a dominant species and insects in an old‐field ecosystem. We further investigate the role of a dominant plant species and insects in shaping ecosystem stability, i.e., the temporal variability of aboveground plant biomass and richness. Specifically, we ask whether there are independent and interactive effects of a dominant plant species and insects on a suite of factors, including aboveground plant biomass, species richness, the community weighted means of plant functional traits, microclimate, and decomposition rate. We additionally ask whether there are independent and interactive effects of a dominant plant species and insects on the ecosystem stability of aboveground plant biomass and species richness.

## 2 Materials and Methods

### 2.1 Site Description

We conducted this experiment in an old field at Matthaei Botanical Garden in Ann Arbor, Michigan (42.30° N, 83.66° W). Local mean annual precipitation is 954 mm (U.S. Climate Data 2024). In 2024, local average monthly temperature ranged from −8°C in January to 29°C in July (Weather Spark 2025). The dominant plant species at this site is *Solidago canadensis* (Canada goldenrod), which on average accounts for ~50% of aboveground plant biomass (Eckberg et al. 2023). *Solidago canadensis* is a perennial forb that ranges across the Americas and is a highly invasive species throughout Europe and Asia (Abrahamson and Weis 1997; Szymura et al. 2016; Zhu et al. 2022). The genus *Solidago* has been studied extensively in both its native and invasive ranges (Abrahamson and Weis 1997; Carson and Root 2000; Szymura et al. 2016; Zhu et al. 2022; Eckberg et al. 2023, 2025), including a number of experiments at this old‐field site that explicitly investigated interactions between *S. canadensis* and associated insects (Eckberg et al. 2023, 2025). Given the global range of *S. canadensis* , the experimental tractability of old‐field plant communities, and previous work at this site, our study site is a system well‐suited to study the effects of dominant plants and insects above‐ and below‐ground as well as over time. This site is mowed semiannually to maintain it in an early‐successional state.

### 2.2 Experimental Design

In August 2021, we organized 24 5 × 5 m experimental plots in a three‐block design, with each block containing eight plots. From summer 2021 to 2024, we maintained a 1‐m walkway among plots by intermittently mowing paths. Within each 5 × 5 m plot, we manipulated the presence of the dominant plant species at two levels in two 1‐m^2^ subplots that were at least 1 m apart: (1) 100% *S. canadensis* removal and (2) control. In *S. canadensis* removal subplots, we removed all *S. canadensis* stems by clipping *S. canadensis* stems at the soil level starting in August 2021. To maintain *S. canadensis* removal, during the growing season from 2022 to 2024 we removed all new *S. canadensis* stems present in *S. canadensis* removal subplots every 3 weeks. We did not remove any plant biomass in control subplots. In addition to *S. canadensis* removal and control subplots, in May 2022 we established one 1‐m^2^ random biomass removal subplot within each 5 × 5 m plot to account for potential biomass removal induced bias (Monteux et al. 2024). Within each 5 × 5 m plot, whenever we removed biomass from *S. canadensis* removal subplots, we removed an equivalent amount of haphazardly selected biomass in the paired random biomass removal subplot, but we removed plants in a non‐species‐specific manner (Monteux et al. 2024). We removed plant biomass in random biomass removal subplots by clipping plants at the soil level. Plant biomass was 36% lower in *S. canadensis* removal subplots (512.9 g m^−2^ ± 266.26) relative to random biomass removal subplots (695.95 g m^−2^ ± 273.4; *t*_paired_ = 2.71, df = 23, *p* = 0.01), suggesting that our results are due to removing *S. canadensis* specifically rather than removing aboveground plant biomass in general.

To study the effect of insects on plant community structure and ecosystem function, we manipulated insect abundance in each 5 × 5 m experimental plot at two levels from 2022 to 2024: (1) insects reduced, and (2) insects present. Starting in May, we reduced insect abundancein 12 of the 24 5 × 5 m plots by spraying a synthetic pyrethroid insecticide, lambda‐cyhalothrin (LamdaStar UltraCap 9.7%; FarmHannong America Inc.), every 3 weeks at a rate of 0.002 L per 1‐m^2^ with a backpack sprayer on sunny days with low wind speed. We calculated spray rate based on the insecticide label instructions for reducing insects in herbaceous plant communities. Pyrethroid insecticides have minimal effects on non‐target terrestrial taxa (Kaneko 2011; Gajendiran and Abraham 2018; Brunk et al. 2019), and while lambda‐cyhalothrin in particular can be toxic to non‐target taxa at high concentrations, the concentration of insecticide that we sprayed falls far below that toxic threshold (Gajendiran and Abraham 2018). When we sampled insects using a leaf blower modified to collect insects, insect abundance was 81% lower in plots sprayed with insecticide (3 ± 2 individuals m^−2^) relative to adjacent areas that were not sprayed with insecticide (16 ± 4 individuals m^−2^; Eckberg et al. 2025). In the 12 5 × 5 m plots where insects were present, we sprayed an equivalent amount of water at the same time as insecticide applications. By manipulating dominant species in 1‐m^2^ subplots within each 5 × 5 m plot, this experiment was a full factorial manipulation that removed a dominant plant species and reduced insect abundance.

### 2.3 Data Collection

To quantify the independent and interactive effects of dominant species removal and insect reduction on the community and ecosystem, we collected the following data: plant species richness, total aboveground plant biomass, subdominant plant biomass, the community‐weighted means of plant functional traits, light availability, soil microclimate, and decomposition rate.

In 2022–2024, we identified all plant species present in each *S. canadensis* removal and control subplot in August (Appendix S1). We then visually estimated the percent cover of each plant species in each subplot. To quantify aboveground plant biomass, we clipped all plant species in a 50 × 20 cm section of each subplot at the soil level. We then sorted all clipped plants into functional groups (i.e., shrub, forb, grass); we also independently recorded the biomass of *S. canadensis* . After sorting plant species, we dried plant samples at 60°C for at least 48 h and then weighed them. To determine total plant biomass in each subplot, we summed the biomass of each plant functional group and *S. canadensis* biomass. To determine subdominant plant biomass in each subplot, we summed the biomass of each plant functional group and excluded *S. canadensis* biomass. Finally, we scaled up all biomass measurements to estimate plant biomass in the entirety of each 1‐m^2^ subplot.

In August 2024, we measured seven plant functional traits: plant height (H), leaf area (LA), specific leaf area (SLA), leaf dry matter content (LDMC), leaf carbon concentration (LCC), leaf nitrogen concentration (LNC), and the ratio of leaf carbon to nitrogen (C:N). We collected trait data in six insect‐reduced and six insect‐present 5 × 5 m plots (12 plots) in both the *S. canadensis* removal and control subplots, for a total of 24 subplots (12 plots × 2 subplots). For each subplot included in the analysis of traits, the total percent cover of all species selected for trait measurements was at least 80% and no more than five species were selected per subplot. For each species selected for trait analysis, we measured the traits of three randomly selected individuals in each subplot. For each individual, we first measured *H* (cm) to the nearest 0.5 cm in the field. We then collected between one and 10 leaves from each individual, wrapped the petiole of each leaf in a wet paper towel, and stored collected leaves separately in plastic bags in the field before putting them in a refrigerator to keep the leaf tissue fresh for additional trait measurements. Within 24 h of collecting leaves, we scanned leaves (Epson Perfection V19) and used the scanned leaf images to calculate LA (cm^2^) using ImageJ (Schneider et al. 2012). Immediately after scanning leaves, we weighed the leaves for each individual to the nearest 0.01 g, oven‐dried the leaves at 60°C, and then re‐weighed them. We calculated mean leaf area by summing the area of all leaves collected for each individual and dividing by the total number of leaves (hereafter referred to as “leaf area”). For both fresh and oven‐dry leaf mass, we calculated the mean leaf mass for each individual by dividing the total mass of all leaves collected by the number of leaves collected. We calculated SLA (cm^2^ g^−1^) as mean leaf area divided by mean oven‐dry leaf mass and LDMC (g g^−1^) as mean oven‐dry leaf mass divided by mean fresh leaf mass. Finally, we homogenized dried leaf samples by species per subplot using a Mini Beadbeater‐96 (BioSpec Products). After grinding leaf samples, we oven‐dried the samples a second time at 60°C in a convection oven. We then packed 0.15 g of each oven‐dried leaf sample into tin capsules and ran the tin capsules through a LECO 628 (LECO Corporation) to determine LCC and LNC (%). We then calculated the ratio of LCC to LNC by dividing LCC by LNC.

To estimate the effects of dominant species removal and insect reduction on the decomposition of soil organic matter, we employed the Tea Bag Index developed by Keuskamp et al. (2013). In each *S. canadensis* removal and control subplot, we buried one Lipton green tea and one Lipton rooibos tea bag in early June 2024. Prior to burying each tea bag, we oven‐dried tea bags at 70°C and weighed each tea bag to the nearest 0.0001 g (Keuskamp et al. 2013). We buried each tea bag at a depth of ~8 cm (Keuskamp et al. 2013). We retrieved tea bags after a 90‐day incubation period (Keuskamp et al. 2013). After recovering the tea bags, we removed any soil particles adhered to the exterior of the tea bag and oven‐dried the tea bags for at least 48 h at 70°C (Keuskamp et al. 2013). We then removed the oven‐dried tea from each tea bag and weighed only the tea to the nearest 0.0001 g (Keuskamp et al. 2013). We estimated the initial weight of the tea in each tea bag by oven‐drying 10 additional tea bags for each tea type at 70°C for at least 48 h and then weighing only the tea bag, string, and label (Keuskamp et al. 2013). For each tea type, we then calculated an average tea bag, string, and label weight and subtracted the mean tea bag, string, and label weight from the initial total tea bag weight estimate to obtain the initial weight of the tea only (Keuskamp et al. 2013). Using the estimates of initial tea weight and the post‐soil incubation tea weight for both the green and rooibos tea, we estimated the decomposition rate using the following equation developed by Keuskamp et al. (2013):

(1) $$k=\ln\left(\mathrm{ar}/\left(W_{t}-\left(1-\mathrm{ar}\right)\right)\right)/t$$

where *k* represents the decomposition rate, *ar* represents the predicted labile fraction of the rooibos tea, *W*_*t*_ represents the weight of the rooibos tea after the incubation time, and *t* represents the incubation time in days. We deployed 96 tea bags total and used 38 to calculate the decomposition fraction (8 *S. canadensis* removal, insects present; 8 *S. canadensis* removal, insects reduced; 10 control, insects present; 12 control, insects reduced). Many of the tea bags we deployed were dug up or damaged, and we did not include these tea bags in our analysis.

To quantify the effects of dominant species removal and insect reduction on light and soil microclimate, we estimated light availability by measuring light intensity at three points above the tallest plants before removing any plant biomass within each 1‐m^2^ subplot in August 2024. We then measured light intensity at three points at ~50 cm above the soil within each subplot. We calculated light availability as mean light intensity at ~50 cm above the soil divided by mean light intensity above the tallest plants. We took all light intensity measurements using the iOS app Lux Light Meter Pro version 2.1.1 (Polyanskaya 2021). To measure belowground soil properties, we deployed one TMS‐4 datalogger (Wild et al. 2019) in each 1‐m^2^ subplot. Each TMS‐4 datalogger measured soil volumetric water content (VWC; %) and soil temperature (°C) at a depth of ~6 cm every 15 min from deployment in the field in September 2023 to the end of September 2024. We calculated mean VWC and mean daytime soil temperature for each subplot from May to September in 2024 for statistical analysis. Importantly, soil properties that we did not measure, such as pH, texture, and nutrients, often vary across small spatial scales (Lin et al. 2005; Xue et al. 2019) and can affect plant community structure and ecosystem function (Whisler et al. 2016; Xu et al. 2025). Though we did not account for spatial variation in soil pH, texture, nutrients, or other soil characteristics in our study, given that our experiment took place in a single field at a fine spatial scale, such variation in such soil factors likely had a limited effect on our results.

### 2.4 Data Analysis

Prior to conducting statistical analyses, we used the plant functional trait measurements to calculate the community weighted mean (CWM) for each trait. We calculated the CWM of each trait in each subplot by weighting the measured trait value by the percent cover of each plant species, which estimated a community‐wide measure for each trait (Lepš and de Bello 2023). We performed the CWM calculations using the *dbFD* function in the “FD” R package (Laliberté and Legendre 2010) in R (R Core Team 2022).

To estimate the temporal stability of total plant biomass and plant species richness, we calculated a coefficient of variation using the following equation:

(2) $$\mathrm{CV}=\frac{\sigma }{\bar{x}}$$

where CV represents the coefficient of variation, *σ* represents the standard deviation of total plant biomass or plant species richness, and $\bar{x}$ represents the mean of total plant biomass or plant species. For each *S. canadensis* removal and control subplot (48 subplots total), we calculated the temporal CV of total plant biomass and plant species richness by first calculating the mean and standard deviation of total plant biomass and species richness for each subplot from 2022 to 2024. We first calculated a temporal CV for each subplot and then calculated a mean temporal CV for each experimental treatment group ( *S. canadensis* removal, insects present; *S. canadensis* removal, insects reduced; Control, insect‐present; Control, insect‐reduced).

For each response variable, we tested for independent and interactive effects of *S. canadensis* removal and insect reduction using two‐factor ANOVAs. We performed all statistical analyses using R version 4.1.3 (R Core Team 2022).

## 3 Results

**FIGURE 1:**
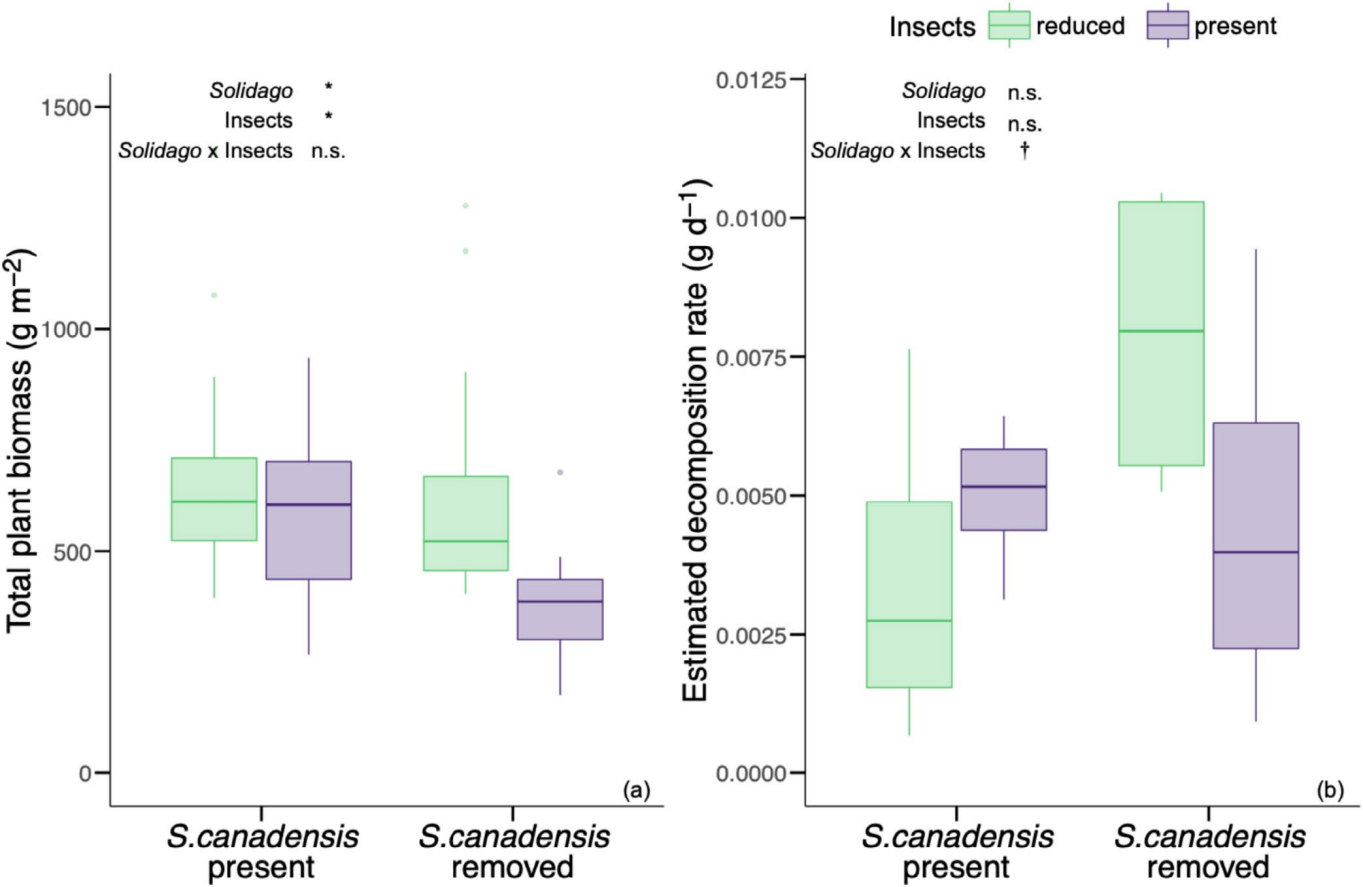
The effect of *S. canadensis* removal and insect reduction treatment on total plant biomass (g m^−2^; a) and the estimated decomposition rate of soil organic matter (b). Green boxplots indicate the mean and interquartile range in insect reduction treatment plots. Purple boxplots indicate the mean and interquartile range in plots where insects were present. Significant effects of *S. canadensis* removal (“*Solidago*”), insect reduction treatment (“Insects”), and their interaction (“*Solidago* × Insects”) are reported as: ***p ≤ 0.001; **p ≤ 0.01; *p ≤ 0.05; †, *p* < 0.1; n.s., not significant.

*Solidago canadensis* removal and insect reduction treatment independently and interactively affected total plant biomass. Total plant biomass was 26% higher in control subplots where *S. canadensis* was present (643.82 g m^−2^ ± 251.76) relative to *S. canadensis* removal subplots (512.9 g m^−2^ ± 266.26; *F* = 4.41_[1,22]_, *p* = 0.047; Figure 1a). Total plant biomass was 37% higher in the insect reduction treatment (668.82 g m^−2^ ± 291.35) relative to where insects were present (487.9 g m^−2^ ± 203.12; *F* = 5.48_[1,22]_, *p* = 0.029; Figure 1a). *Solidago canadensis* removal and insect reduction treatment had no significant interactive effect on estimated decomposition rate (*F* = 3.78_[1,15]_, *p* = 0.07; Figure 1b).

**FIGURE 2:**
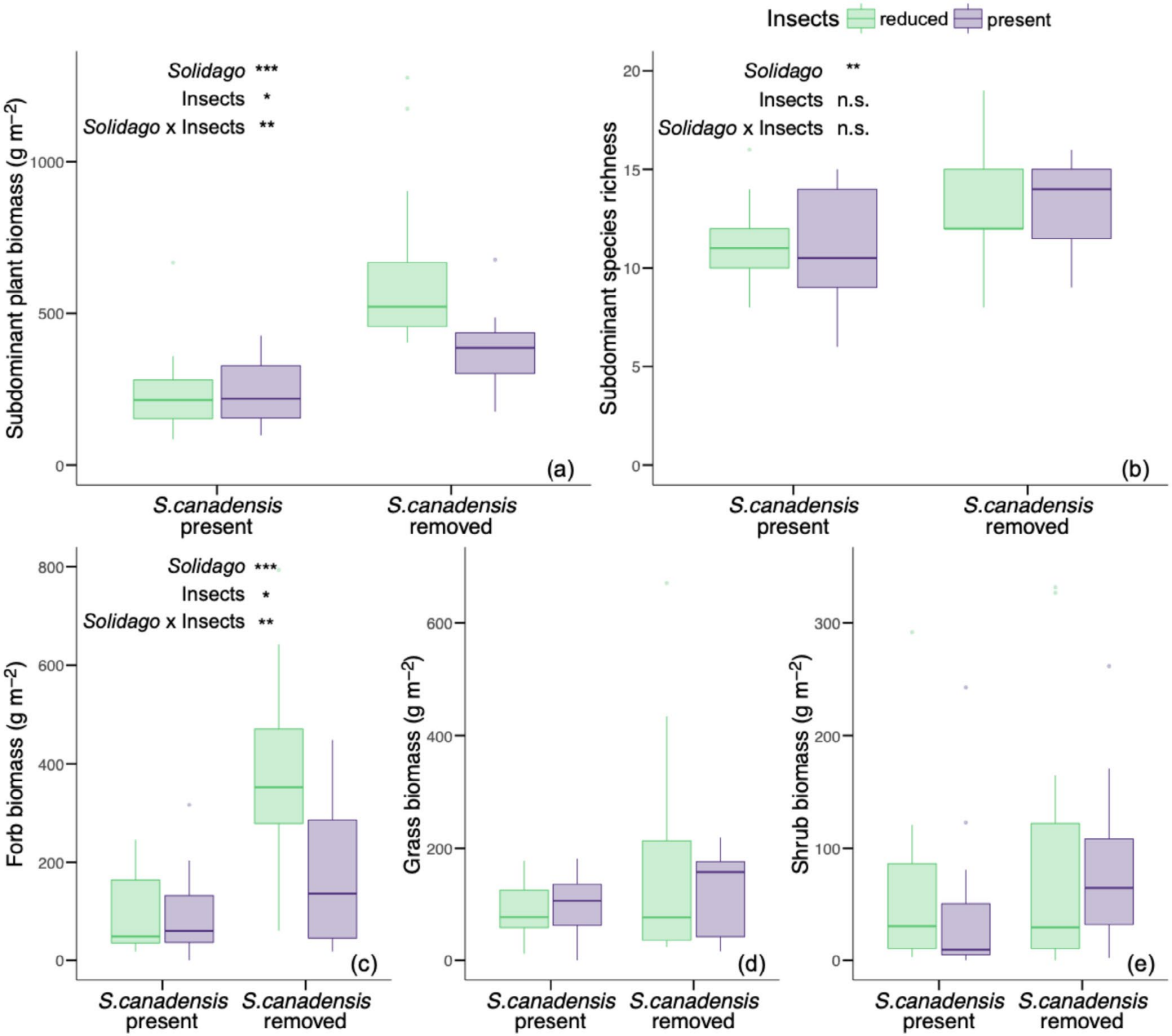
The effect of *S. canadensis* removal and insect reduction treatment on total subdominant plant biomass (g m^−2^; a), subdominant plant species richness (b), subdominant forb biomass (g m^−2^; c), subdominant grass biomass (g m^−2^; d), and subdominant shrub biomass (g m^−2^; e). Green boxplots indicate the mean and interquartile range in insect reduction treatment plots. Purple boxplots indicate the mean and interquartile range in plots where insects were present. Significant effects of *S. canadensis* removal (“*Solidago*”), insect reduction treatment (“Insects”), and their interaction (“*Solidago* × Insects”) are reported as: ***p ≤ 0.001; **p ≤ 0.01; *p ≤ 0.05; †, *p* < 0.1; n.s., not significant.

*Solidago canadensis* removal and insect reduction treatment altered total subdominant plant biomass, subdominant forb biomass, and subdominant species richness, but not subdominant grass or shrub biomass. Total subdominant plant biomass was higher where *S. canadensis* was removed relative to control subplots where *S. canadensis* was present (*F* = 40.05_[1,22]_, *p* < 0.001; Figure 2a) and was higher in the insect reduction treatment relative to where insects were present (*F* = 4.5_[1,22]_, *p* = 0.045; Figure 2a). Specifically, total subdominant plant biomass was 111% higher in *S. canadensis* removal subplots (513.06 g m^−2^ ± 266.11) compared to control subplots where *S. canadensis* was present (243.39 g m^−2^ ± 132.3) and 45% higher in the insect reduction treatment (447.3 g m^−2^ ± 311.36) relative to where insects were present (309.14 g m^−2^ ± 139.12). *Solidago canadensis* removal and insect reduction treatment interactively affected total subdominant plant biomass such that total subdominant plant biomass was highest where *S. canadensis* was removed in the insect reduction treatment (*F* = 9.3_[1,22]_, *p* = 0.006; Figure 2a). Subdominant forb biomass was higher in *S. canadensis* removal subplots relative to control subplots where *S. canadensis* was present (*F* = 27.61_[1,22]_, *p* < 0.001; Figure 2c) and was higher in the insect reduction treatment relative to where insects were present (*F* = 5.65_[1,22]_, *p* = 0.027; Figure 2c). Specifically, subdominant forb biomass was 196% higher in *S. canadensis* removal subplots (278.48 g m^−2^ ± 201.3) relative to control subplots where *S. canadensis* was present (94.21 g m^−2^ ± 84.06), and 77% higher in the insect reduction treatment (238.23 g m^−2^ ± 209.65) relative to where insects were present (134.46 g m^−2^ ± 125.42). *Solidago canadensis* removal and insect reduction treatment interactively affected subdominant forb biomass such that subdominant forb biomass was highest in *S. canadensis* removal subplots in the insect reduction treatment (*F* = 9.3_[1,22]_, *p* = 0.006; Figure 2c). *Solidago canadensis* removal had a significant effect on subdominant species richness, such that subdominant species richness was 19% higher in *S. canadensis* removal subplots (13.25 ± 2.63) relative to control subplots where *S. canadensis* was present (11.13 ± 2.52; *F* = 7.83_[1,22]_, *p* = 0.008; Figure 2b).

**FIGURE 3:**
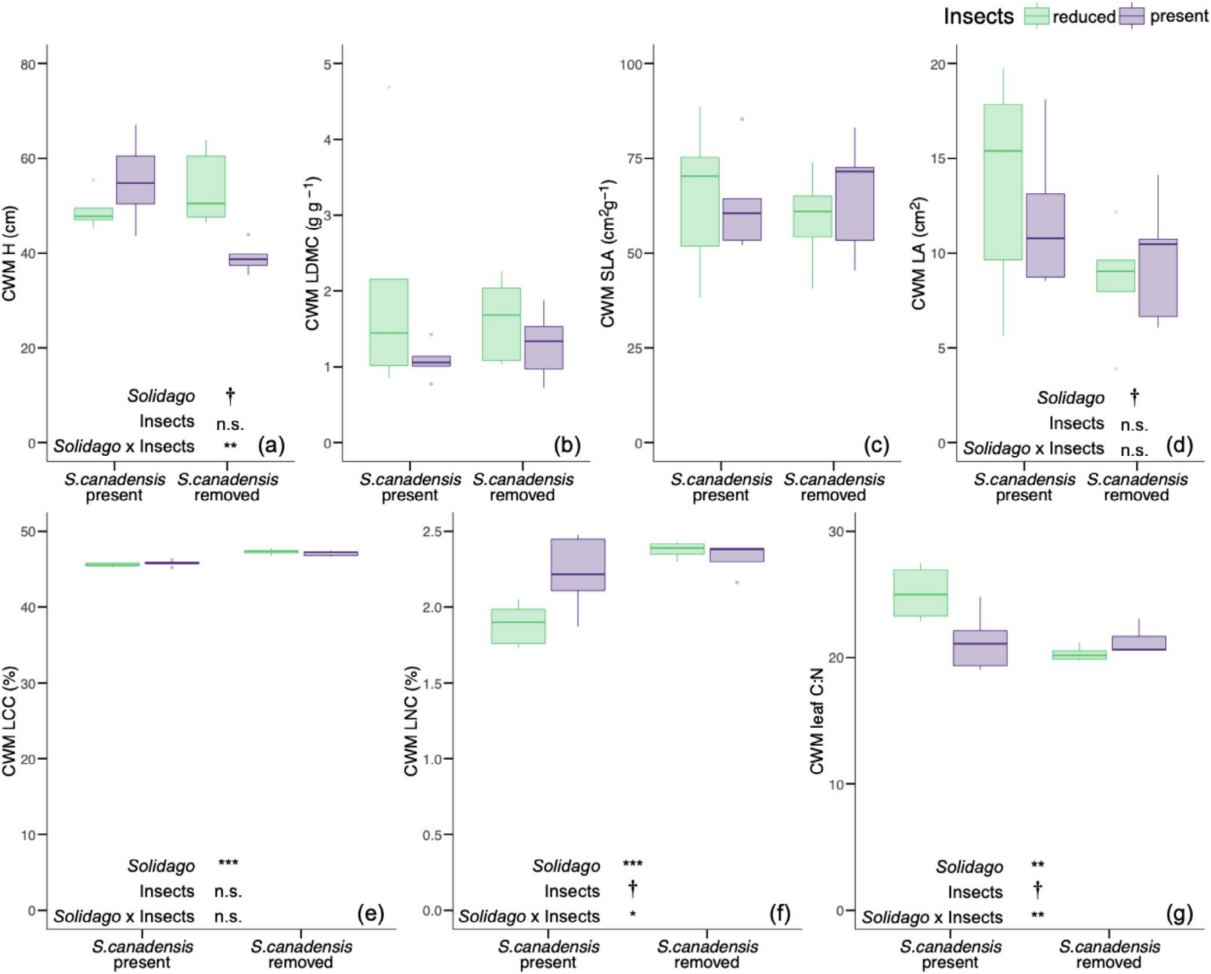
The effect of *S. canadensis* removal and insect reduction treatment on the community weighted mean (CWM) of plant height (H; cm; a), leaf dry matter content (LDMC; g g^−1^; b), specific leaf area (SLA; cm^2^ g^−1^; c), leaf area (cm^2^; d), leaf carbon concentration (LCC; %; e), leaf nitrogen concentration (LNC; %; f), and the ratio of leaf carbon to nitrogen (C:N; g). Green boxplots indicate the mean and interquartile range in insect reduction treatment plots. Purple boxplots indicate the mean and interquartile range in plots where insects were present. Significant effects of *S. canadensis* removal (“*Solidago*”), insect reduction treatment (“Insects”), and their interaction (“*Solidago* × Insects”) are reported as: ***p ≤ 0.001; **p ≤ 0.01; *p ≤ 0.05; †, *p* < 0.1; n.s., not significant.

*Solidago canadensis* removal and insect reduction treatment affected the community weighted mean (CWM) of plant height (*H*) and leaf nutrient concentrations (LCC, LNC), but not leaf area (LA), leaf dry matter content (LDMC), or specific leaf area (SLA). *Solidago canadensis* removal had no independent effect on the CWM of *H* (*F* = 3.89_[1,16]_, *p* = 0.066; Figure 3a). *Solidago canadensis* removal and insect reduction treatment interactively affected the CWM of H such that H was highest where *S. canadensis* was removed in the insect reduction treatment (*F* = 14.92_[1,16]_, *p* = 0.002; Figure 3a). The CWM of LCC was 3% higher in *S. canadensis* removal subplots (47.19% ± 0.37) relative to control subplots where *S. canadensis* was present (45.67% ± 0.31; *F* = 99.65_[1,16]_, *p* < 0.001; Figure 3e). Similarly, the CWM of LNC was 14% higher in *S. canadensis* removal subplots (2.35% ± 0.08) relative to control subplots where *S. canadensis* was present (2.05% ± 0.26; *F* = 18.4_[1,16]_, *p* < 0.001; Figure 3f). Insect reduction treatment had no effect on the CWM of LNC (*F* = 4.32_[1,16]_, *p* = 0.054; Figure 3f). *Solidago canadensis* removal and insect reduction treatment interactively affected the CWM of LNC such that LNC was higher where insects were present only in control subplots where *S. canadensis* was present (*F* = 8.03_[1,16]_, *p* = 0.01; Figure 3f). *Solidago canadensis* removal and insect reduction treatment also had independent and interactive effects on the CWM of leaf C:N. The CWM of leaf C:N was 12% higher in control subplots where *S. canadensis* was present (23.2% ± 2.9) relative to *S. canadensis* removal subplots (20.81% ± 0.98; *F* = 10.17_[1,16]_, *p* = 0.006; Figure 3g). Insect reduction treatment had no effect on the CWM of leaf C:N (*F* = 3.56_[1,16]_, *p* = 0.077; Figure 3g). *S. canadensis* removal and insect reduction treatment had a significant interactive effect on the CWM of leaf C:N such that C:N was higher where insects were present only in control subplots where *S. canadensis* was present (*F* = 10.35_[1,16]_, *p* = 0.005; Figure 3g). *Solidago canadensis* removal had no independent effect on the CWM of LA (*F* = 3.85_[1,16]_, *p* = 0.067; Figure 3d). Finally, there was no effect of *Solidago canadensis* removal (SLA: *F* = 0.08_[1,16]_, *p* = 0.79; LDMC: *F* = 0.07_[1,16]_, *p* = 0.8), insect reduction treatment (SLA: *F* = 0.11_[1,16]_, *p* = 0.75; LDMC: *F* = 2.73_[1,16]_, *p* = 0.12), or their interaction (SLA: *F* = 0.32_[1,16]_, *p* = 0.58, Figure 3c; LDMC: *F* = 0.63_[1,16]_, *p* = 0.44, Figure 3b) on the CWM of SLA or LDMC.

**FIGURE 4:**
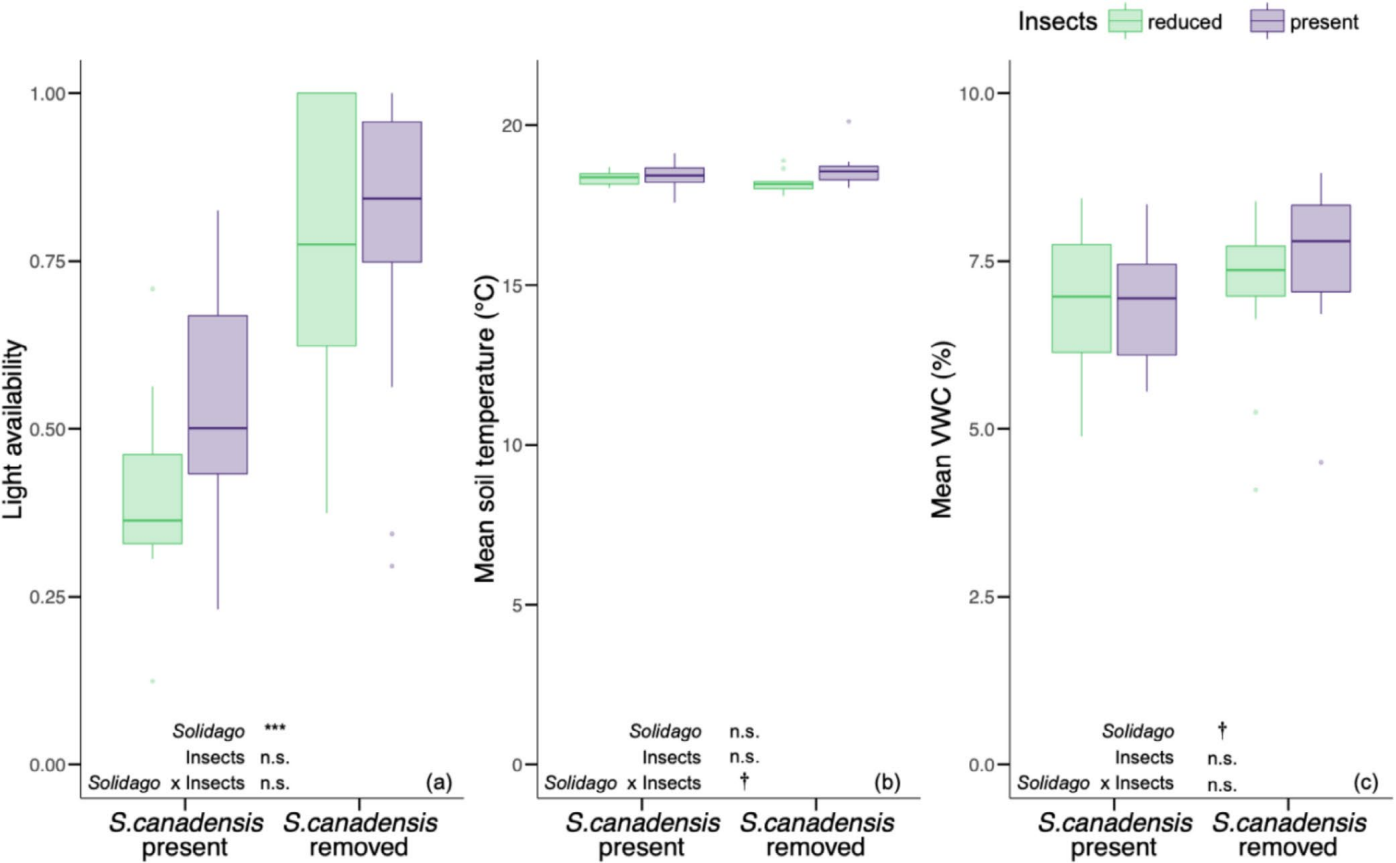
The effect of *S. canadensis* removal and insect reduction treatment on light availability (a), mean soil temperature (°C; b), and mean soil volumetric water content (VWC; %; c). Green boxplots indicate the mean and interquartile range in insect reduction treatment plots. Purple boxplots indicate the mean and interquartile range in plots where insects were present. Significant effects of *S. canadensis* removal (“*Solidago*”), insect reduction treatment (“Insects”), and their interaction (“*Solidago* × Insects”) are reported as: ***p ≤ 0.001; **p ≤ 0.01; *p ≤ 0.05; †, *p* < 0.1; n.s., not significant.

*Solidago canadensis* removal systematically altered abiotic conditions above‐ and below‐ground; the effects of insect reduction treatment on abiotic factors were more limited. In particular, light availability was 64% higher in *S. canadensis* removal subplots (0.78 ± 0.23) relative to control subplots (0.47 ± 0.18; *F* = 32.25_[1,22]_, *p* < 0.001; Figure 4a). *Solidago canadensis* removal and insect reduction treatment did not interactively affect mean soil temperature (°C; *F* = 3.92_[1,22]_, *p* = 0.06; Figure 4b). *Solidago canadensis* removal did not affect mean soil volumetric water content (VWC; %) (*F* = 3.97_[1,22]_, *p* = 0.059; Figure 4c).

**FIGURE 5:**
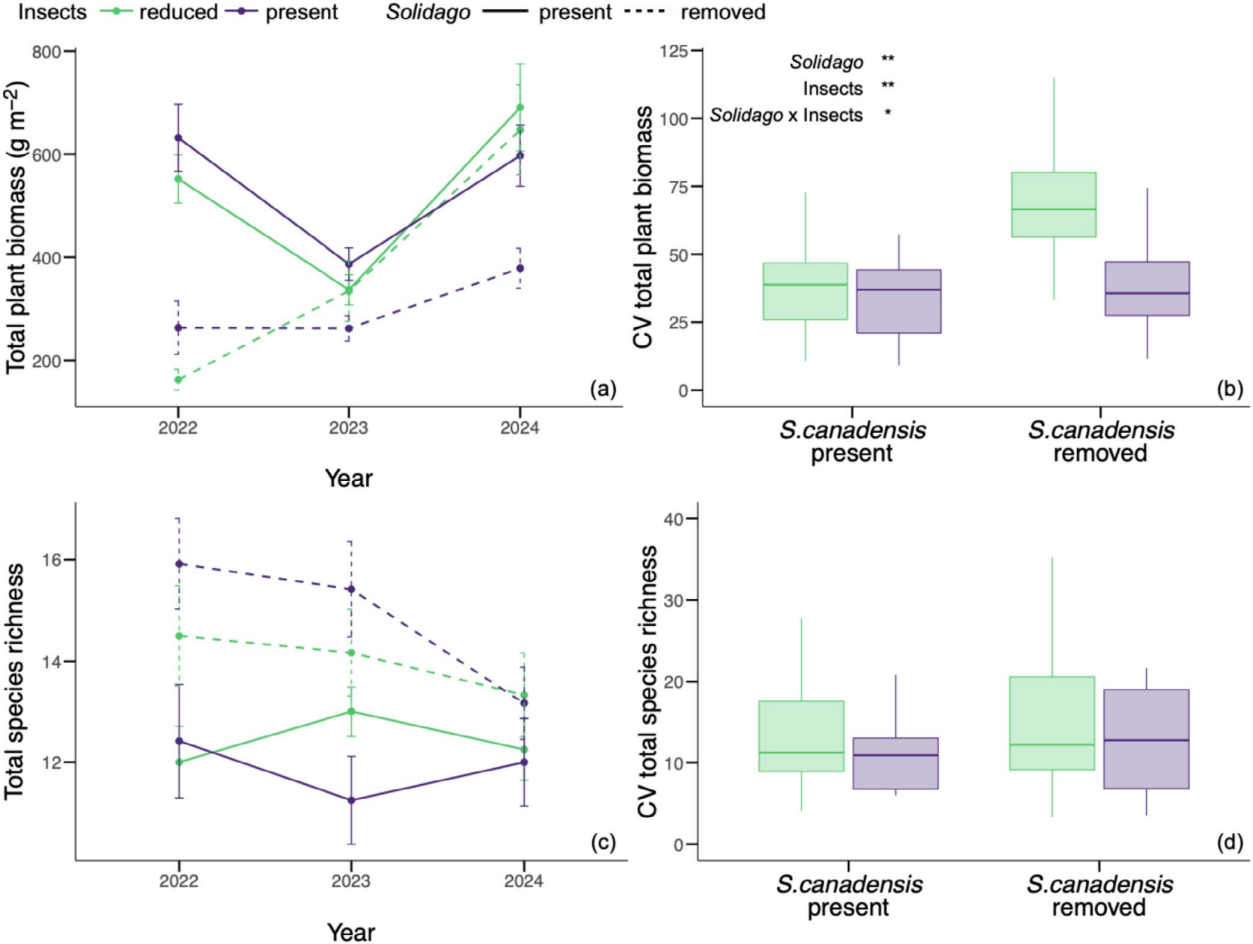
The effect of *S. canadensis* removal and insect reduction treatment on the mean and standard error of total plant biomass (g m^−2^; a) and total species richness (c) from 2022 to 2024. Green points and error bars indicate the yearly mean and standard error in insect reduction treatment plots, and purple points and error bars indicate the yearly mean and standard error in plots where insects were present. Solid lines connect mean total plant biomass and mean total richness of control subplots where *S. canadensis* is present across years, and dashed lines connect mean total plant biomass and mean total richness of *S. canadensis* removal subplots across years. The effect of *S. canadensis* removal and insect reduction treatment on the coefficient of variation (CV) of total plant biomass (b) and total plant species richness (d). Green boxplots indicate the mean and interquartile range of CV in insect reduction treatment plots. Purple boxplots indicate the mean and interquartile range of CV variable in plots where insects were present. Significant effects of *S. canadensis* removal (“*Solidago*”), insect reduction treatment (“Insects”), and their interaction (“*Solidago* × Insects”) are reported as: ***p ≤ 0.001; **p ≤ 0.01; *p ≤ 0.05; †, *p* < 0.1; n.s., not significant.

*Solidago canadensis* removal and insect reduction treatment altered the temporal stability of total plant biomass but not species richness. The coefficient of variation (CV) of total plant biomass was 49% higher in *S. canadensis* removal subplots (54.03 ± 26.4) relative to control subplots where *S. canadensis* was present (36.25 ± 17.21; *F* = 10.37_[1,22]_, *p* = 0.004; Figure 5b). The CV of total plant biomass was also 51% higher in the insect reduction treatment (54.3 ± 26.44) relative to where insects were present (36 ± 16.85; *F* = 10.25_[1,22]_, *p* = 0.004; Figure 5b). *Solidago canadensis* removal and insect reduction treatment interactively affected the CV of total plant biomass such that the CV of total plant biomass was highest in *S. canadensis* removal subplots in the insect reduction treatment (*F* = 5.85_[1,22]_, *p* = 0.02; Figure 5b).

## 4 Discussion

Here, we show that a dominant plant species and insects have independent and interactive effects above‐ and below‐ground in an old‐field ecosystem. Specifically, total plant biomass, a proxy for productivity in annual plant communities, was higher where *S. canadensis* was present and where insects were reduced. Since dominant plant species typically have the largest biomass in a community (Grime 1998; Avolio et al. 2019), it follows that total biomass would be higher where *S. canadensis* was present. Another study on *Solidago* found that total plant biomass was higher where *Solidago* biomass was higher (Carson and Root 1999), emphasizing the role of dominant *Solidago* spp. in shaping productivity in particular. Similarly, given that insect herbivores typically reduce aboveground plant biomass (Carson and Root 1999; Kozlov and Zvereva 2018; Smith et al. 2020; Agrawal and Maron 2022), it follows that total plant biomass would be higher where insects were reduced relative to where insects were present. Overall, *S. canadensis* and insects had opposing effects on productivity in that *S. canadensis* promoted plant biomass while insects reduced plant biomass. Therefore, any explanation of the ecological processes that govern productivity in terrestrial ecosystems should consider the competitive effects of dominant plants and the consumptive effects of insects in combination.

In contrast to their effects on total plant biomass, *S. canadensis* and insects interactively affected the decomposition rate of soil organic matter, such that the decomposition rate of soil organic matter was highest where *S. canadensis* was removed, and insects were reduced. Dominant plants can indirectly affect decomposition rate by modifying the composition and function of the soil microbial communities that decompose organic matter (Zhang et al. 2009; Vaieretti et al. 2018; Wang et al. 2018; Zubek et al. 2020; Yang et al. 2021). For example, dominant plants can reduce water availability (Zubek et al. 2020), and decomposition rate decreases with decreased soil moisture (Lee et al. 2014). Here, soil volumetric water content (VWC) was higher where *S. canadensis* was removed, suggesting that *S. canadensis* could be affecting the decomposition rate of organic matter by modifying water availability. Unlike *S. canadensis* , insects had limited effects on soil microclimate in our study. Therefore, we suggest that insects could be shaping the decomposition rate of soil organic matter through their effects on soil microbes (Yang and Gratton 2014), though we did not explicitly examine this. Furthermore, insects could modify soil microbial community composition or function indirectly by modifying plant traits. For example, leaf nitrogen concentration (LNC) positively correlates with litter decomposition rate (Van Der Heijden et al. 2008; Bakker et al. 2011). Here, we find that insects modify LNC, suggesting that through their effects on the quality of leaf litter inputs, insects may indirectly reshape soil microbe function and ultimately modify the rate of decomposition. The capacity for insects to affect decomposition through effects of soil microbes, along with the positive effect of *S. canadensis* removal on soil VWC, suggests that the decomposition rate of soil organic matter would be highest where *S. canadensis* was removed, and insects were reduced.

In addition to shaping above‐ and below‐ground ecosystem function, dominant species and insects affected total subdominant plant biomass, particularly the biomass of subdominant forbs. Total subdominant plant biomass and the biomass of subdominant forbs were both higher where *S. canadensis* was removed, where insects were reduced, and was highest in *S. canadensis* removal subplots, where insects were reduced. We previously found that total subdominant plant biomass was higher where *S. canadensis* biomass was lower (Eckberg et al. 2023) and that *S. canadensis* and insect herbivores interactively shape subdominant plant biomass (Eckberg et al. 2025). We expand on this previous work by showing that the relatively higher subdominant plant biomass in *S. canadensis* removal subplots was largely driven by the higher biomass of subdominant forbs where *S. canadensis* was removed. The positive effect of *S. canadensis* removal on subdominant forbs could be explained by the limiting similarity hypothesis, which posits that species that are functionally similar are less likely to stably coexist (MacArthur and Levins 1967; Schamp and Jensen 2019; Munoz et al. 2023). Since *S. canadensis* is also a forb, we suggest that the removal of *S. canadensis* would particularly benefit functionally similar subdominant forbs. Given that dominant plants reduce the biomass of subdominant plants (Avolio et al. 2019; Eckberg et al. 2023, 2025) and insect herbivores reduce plant biomass overall (Carson and Root 1999; Smith et al. 2020), it follows that the biomass of subdominant plants would then be highest where *S. canadensis* was removed, and insects were reduced.

*Solidago canadensis* removal, but not insect reduction, affected subdominant species richness, wherein subdominant richness was higher where *S. canadensis* was removed relative to where *S. canadensis* was present. Other field experiments similarly found that removing a dominant plant species from a community increased plant richness (Souza et al. 2011; Avolio et al. 2019; Smith et al. 2020), emphasizing the negative effect of dominant plants on plant diversity (Zhang et al. 2025). Though insect herbivores can have positive effects on plant richness (Carson and Root 1999; Smith et al. 2020; Agrawal and Maron 2022), a recent meta‐analysis reported that the effect of insect reduction on plant richness was lower in magnitude than the effect of dominant species removal on plant richness (Smith et al. 2020). A difference in the magnitude of effect of *S. canadensis* and insects on plant richness could result in no detectable effect of insects on plant richness in our study.

*Solidago canadensis* likely affected the biomass and richness of subdominant plants by reducing light availability and soil VWC. Competition for light is an important driver of community structure and ecosystem function (Vojtech et al. 2008; Hautier et al. 2009, 2018; Borer et al. 2017), as the ability of plant species to intercept light can determine the competitive advantage of a species in productive environments (Hautier et al. 2018). Light availability was higher where *S. canadensis* was removed, suggesting that *S. canadensis* , in part, reduced the biomass and richness of subdominant plants by outcompeting subdominant species for light. Our work is in line with previous studies that show that dominant plants can shape the biomass and richness of plant communities by modifying the light environment (Emery and Gross 2007; McCain et al. 2010; Eckberg et al. 2023). Similarly to competition for light, competition for water can also affect plant community structure (Craine and Dybzinski 2013; Schwinning and Kelly 2013; Xu et al. 2013). Here, soil VWC was higher where *S. canadensis* was removed, suggesting that competition with *S. canadensis* for both water and light reduced subdominant plant biomass and richness.

*Solidago canadensis* and insects affected the community weighted mean (CWM) of several plant traits, including plant height (*H*) and leaf nutrient concentrations. The CWM of H and the ratio of leaf carbon to nitrogen (C:N) were higher where *S. canadensis* was present compared to where *S. canadensis* was removed, and leaf area (LA) was marginally higher in control subplots where *S. canadensis* was present. Importantly, in our analysis, we measured traits of subdominant plant species. As such, since leaf C:N negatively correlates with carbon assimilation and growth rate capacities (Sheng et al. 2021), the growth rate capacity of subdominant plants was likely lower where *S. canadensis* was present, and light was limited. Plant height and leaf area are also associated with light competition (Pérez‐Harguindeguy et al. 2013), and plants typically increase investment in height and leaf area to maximize light capture when light is limited (Wright et al. 2004; Chen et al. 2010). In our study, subdominant plants could invest more in growing taller and producing larger leaves to better compete for light where *S. canadensis* was present.

In contrast, leaf nitrogen concentration (LNC) and leaf carbon concentration (LCC) were higher where *S. canadensis* was removed, though only LNC was affected by insects. Importantly, LNC can positively correlate with photosynthetic rate (Pérez‐Harguindeguy et al. 2013; Peng et al. 2021). Since light availability was higher where *S. canadensis* was removed, subdominant plants could invest more in photosynthetic capacity where light was not limited, and photosynthetic rate could be maximized. Insect presence also affected LNC such that LNC was higher where insects were present. Since many defense metabolites contain nitrogen (Singh 2018; Wang et al. 2022; Li et al. 2024), subdominant plants could have invested more in nitrogen‐based defenses where insects were present. Though there was no effect of insects on LCC, if photosynthetic capacity was in fact greater where *S. canadensis* was removed, LCC could have been higher, as more carbon would have been assimilated (Dusenge et al. 2019).

Notably, *S. canadensis* and insects interactively affected the CWM of H, leaf C:N, and LNC. In particular, plants were taller where insects were reduced only where *S. canadensis* was removed. We posit that the effect of insects on plant height was only detectable where *S. canadensis* was removed. A recent meta‐analysis found that the effect of dominant species removal on plant productivity and species richness exceeded the effects of 11 other global change drivers, including insect reduction (Smith et al. 2020), emphasizing the sheer magnitude of the effect of dominant plants on communities and ecosystems. Therefore, we suggest that the CWM of H was higher where insects were reduced only where *S. canadensis* was removed because the relatively stronger selective effect of *S. canadensis* on plant height masked any effect of insects. In contrast, leaf C:N was higher where insects were reduced only where *S. canadensis* was present. If subdominant plants have lower photosynthetic capacities when competing with a dominant plant species that reduces light availability (Sheng et al. 2021), leaf C:N could ultimately have been higher where *S. canadensis* was present and insects were reduced. Furthermore, LNC was higher where insects were present only where *S. canadensis* was present, which could have further contributed to the overall higher leaf C:N where *S. canadensis* was present and insects were reduced.

Finally, the temporal stability of plant biomass, but not species richness, was affected by *S. canadensis* and insects. The coefficient of variation (CV) of total plant biomass was higher where *S. canadensis* was removed, where insects were reduced, and was highest where *S. canadensis* was removed and insects were reduced. The stability of plant biomass tends to be higher where plant biomass is high (Isbell et al. 2009; Yang et al. 2017), suggesting that where *S. canadensis* was present, and total plant biomass was higher, plant biomass would be less variable over time. Furthermore, since dominant plants govern ecosystem processes (Grime 1998), if *S. canadensis* was biomass stable over time, the community would then be more stable and have a lower CV where *S. canadensis* was present. However, the stability of biomass was not driven solely by plant biomass because even though biomass was higher where insects were reduced, biomass stability was lower. We suggest that the response of subdominant plants to *S. canadensis* removal and insect reduction resulted in lower stability and a higher temporal CV. In other words, where we removed *S. canadensis* and insects, independently and in combination, plant biomass increased over time. When plant biomass increased over time, there was greater variability in biomass and an overall higher CV.

Importantly, we found that subdominant plant compensation for *S. canadensis* removal occurred only where insects were reduced. Compensation is the degree to which productivity is recovered by remaining plant species in a community following plant species loss (Adler and Bradford 2002; McLaren and Turkington 2011). After 3 years of *S. canadensis* removal and insect reduction, total plant biomass where *S. canadensis* was removed and insects were reduced had recovered to the same amount of total biomass where *S. canadensis* was present. Critically, where *S. canadensis* was removed and insects were present, total plant biomass did not recover to the amount of biomass where *S. canadensis* was present. Taken together, we suggest that through their top‐down consumptive effects, insect herbivores limit the recovery of productivity following dominant species loss. We further emphasize the necessity of considering the effects of insect herbivores when trying to understand and predict the response of terrestrial plant communities to biodiversity loss over time.

Our study underscores the interactive role of a dominant plant species and insects in structuring plant communities, altering microclimate, shaping ecosystem function above‐ and below‐ground, and perhaps even the recovery or restoration of communities. Whether the effects of insect herbivores extend belowground remains a relatively unexplored area of community ecology, and we show that the effect of insects on soil microclimate and decomposition is mediated by a dominant plant species. Future work should further investigate the mechanisms by which insects shape the biomass, traits, and stability of herbaceous plant communities. Given the limited effects of insects on abiotic conditions in this study, future studies should directly test if insect presence affects the composition or function of soil microbes as a potential biotic mechanism of insect control of terrestrial plant community structure and biomass.

Furthermore, we show that dominant plants and insects shape the temporal stability of plant biomass and emphasize the necessity of tracking the effects of experimental field manipulations on communities and ecosystems over multiple years. Insect herbivores are clearly important mediators of plant community recovery after biodiversity loss, and amid accelerating species loss and insect decline (Bellard et al. 2012; Goulson 2019), our results underscore the necessity of integrating dominant plant and insect herbivore interactions in order to predict how terrestrial communities may be impacted by global change. Overall, we highlight the need to consider the potential interactive effects of dominant plants and insects above‐ and below‐ground, as well as through time, in order to improve our understanding of their role structuring communities and ecosystems.

## Author Contributions

**Julia N. Eckberg:** conceptualization (lead), data curation (lead), formal analysis (lead), funding acquisition (lead), investigation (lead), methodology (lead), project administration (lead), visualization (lead), writing – original draft (lead), writing – review and editing (lead). **Nathan J. Sanders:** conceptualization (supporting), data curation (supporting), formal analysis (supporting), funding acquisition (supporting), investigation (supporting), methodology (supporting), project administration (supporting), supervision (lead), visualization (supporting), writing – original draft (supporting), writing – review and editing (supporting).

## Conflicts of Interest

The authors declare no conflicts of interest.

## Supporting information


**Appendix S1:** ece372742‐sup‐0001‐AppendixS1.docx.

## Data Availability

Data are available from Dryad. DOI: https://doi.org/10.5061/dryad.18931zd8x.
