# Supplementary material for: A Dominant Plant Species and Insects Independently and Interactively Shape Plant Community Structure and Ecosystem Function Above‐ and Below‐Ground
Source: Ecol Evol. 2025 Dec 19;15(12):e72742. doi: 10.1002/ece3.72742 (PMC12717143; doi:10.1002/ece3.72742)
Supplement: Supplementary file 1 — Appendix S1: ece372742‐sup‐0001‐AppendixS1.docx. [file ECE3-15-e72742-s001.docx]

Ecology and Evolution

**A dominant plant species and insect herbivores independently and interactively shape plant community structure and ecosystem function above- and below-ground**

Julia N. Eckberg & Nathan J. Sanders

**Appendix S1**

**Table S1**: List of all plant species present at the study site in August 2024 from the most abundant species to the least abundant species. Excluded from the table is an unidentified species in the family Asteraceae which is the 37^th^ most abundant species at this site after Asclepias syriaca.

| **Genus** | **Species** |
| --- | --- |
| *Solidago* | *canadensis* |
| *Poa* | *pratensis* |
| *Monarda* | *fistulosa* |
| *Hypericum* | *perforatum* |
| *Galium* | *verum* |
| *Rubus* | *occidentalis* |
| *Vitis* | *riparia* |
| *Pilosella* | *longifolia* |
| *Parthenocissus* | *quinquefolia* |
| *Toxicodendron* | *rydbergii* |
| *Euthamia* | *graminifolia* |
| *Apocynum* | *cannabinum* |
| *Cornus* | *racemosa* |
| *Origanum* | *vulgare* |
| *Symphyotrichum* | *drummondii* |
| *Rhamnus* | *cathartica* |
| *Daucus* | *carota* |
| *Cirsium* | *arvense* |
| *Frangula* | *alnus* |
| *Panicum* | *dichotomiflorum* |
| *Geum* | *macrophyllum* |
| *Rosa* | *blanda* |
| *Linaria* | *vulgaris* |
| *Symphyotrichum* | *lateriflorum* |
| *Achillea* | *millefolium* |
| *Sorghastrum* | *nutans* |
| *Tanacetum* | *vulgare* |
| *Potentilla* | *recta* |
| *Erigeron* | *strigosus* |
| *Leucanthemum* | *vulgare* |
| *Digitalis* | *grandiflora* |
| *Rosa* | *blanda* |
| *Fraxinus* | *americana* |
| *Digitalis* | *grandiflora* |
| *Fragaria* | *virginiana* |
| Asclepias | syriaca |
| *Taraxacum* | *officinale* |
| *Geum* | *aleppicum* |
| *Malus* | *fusca* |
| *Galium* | *triflorum* |
| *Vicia* | *tetrasperma* |
| *Carduus* | *acanthoides* |
| *Cerastium* | *fontanum* |
| *Lactuca* | *canadensis* |
| *Ranunculus* | *sp.* |
| *Rumex* | *acetosella* |
| *Morus* | *alba* |
